# Identification of small effect quantitative trait loci of plant architectural, flowering, and early maturity traits in reciprocal interspecific introgression population in cotton

**DOI:** 10.3389/fpls.2022.981682

**Published:** 2022-08-18

**Authors:** Rahul Chandnani, Changsoo Kim, Jinesh D. Patel, Hui Guo, Tariq Shehzad, Jason G. Wallace, Daohua He, Zhengsheng Zhang, Jeevan Adhikari, Sameer Khanal, Peng W. Chee, Andrew H. Paterson

**Affiliations:** ^1^Plant Genome Mapping Laboratory, The University of Georgia, Athens, GA, United States; ^2^Department of Crop Science, College of Agriculture and Life Sciences, Chungnam National University, Daejeon, South Korea; ^3^Department of Crop and Soil Sciences, University of Georgia, Athens, GA, United States; ^4^College of Agronomy, Northwest A&F University, Yangling, Shaanxi, China; ^5^College of Agronomy and Biotechnology, Southwest University, Chongqing, China; ^6^NESPAL Molecular Cotton Breeding Laboratory, The University of Georgia, Tifton, GA, United States

**Keywords:** quantitative trait loci, introgression, cotton, plant height, maturity, flowering

## Abstract

Plant architecture, flowering time and maturity traits are important determinants of yield and fiber quality of cotton. Genetic dissection of loci determining these yield and quality components is complicated by numerous loci with alleles conferring small differences. Therefore, mapping populations segregating for smaller numbers and sizes of introgressed segments is expected to facilitate dissection of these complex quantitative traits. At an advanced stage in the development of reciprocal advanced backcross populations from crosses between elite *Gossypium hirsutum* cultivar ‘*Acala Maxxa*’ (GH) and *G. barbadense* ‘*Pima S6*’ (GB), we undertook mapping of plant architectural traits, flowering time and maturity. A total of 284 BC_4_F_1_ and BC_4_F_2_ progeny rows, 120 in GH and 164 in GB background, were evaluated for phenotype, with only 4 and 3 (of 7) traits showing significant differences among progenies. Genotyping by sequencing yielded 3,186 and 3,026 SNPs, respectively, that revealed a total of 27 QTLs in GH background and 22 in GB, for plant height, days to flowering, residual flowering at maturity and maturity. More than of 90% QTLs identified in both backgrounds had small effects (%PV < 10), supporting the merit of this population structure to reduce background noise and small effect QTLs. Germplasm developed in this study may serve as potential pre-breeding material to develop improved cotton cultivars.

## Introduction

Plant architecture, the three-dimensional spatial arrangement of plant parts, reflects how plants allocate resources to different parts such as branches, flowers, fruits, etc. (Kahlen et al., 2015). Different architectures facilitate growth and productivity in particular environments (Reinhardt and Kuhlemeier, 2002), and make crop specific agronomic practices efficient (Song and Zhang, 2009). Plant architecture of most economically important crops has been affected by domestication (Clark et al., 2006; Jin et al., 2008; Cai et al., 2016) and selection for yield and quality improvement, for example, selection for ‘dwarf’ rice and wheat varieties during the Green Revolution resulted in significant increases in harvestable grain yield by increasing resistance to lodging and permitting increased fertilization (Peng et al., 1999). Another important aspect of plant architecture is flowering time, the timing of the transition from vegetative to reproductive growth, with different growing conditions necessitating different timing to attain high yield and quality (Peng et al., 1999; Tasma et al., 2001).

Plant architectural traits are complex, influenced by environment but also known to be controlled genetically (Song and Zhang, 2009) and have been analyzed and/or genetically mapped in plants ranging from models such as *Arabidopsis thaliana* to commercially important crops like cereals (maize, wheat, rice), soybean, tomato and cotton (Hareven et al., 1996; Bradley et al., 1997; Kulwal et al., 2003; Deng et al., 2006; Wang and Li, 2006; Liu et al., 2007; Sang and Ge, 2007; Song and Zhang, 2009).

Cotton is one of the most economically important cash crops and the most widely used textile fiber crop. An estimated 12.2 million acres of land in the United States was planted to cotton in 2022 (USDA-NASS, 2022). Cotton belongs to the genus *Gossypium* and there are presently about 45 diploids and seven allotetraploid species recognized in this genus (Smith and Cothren, 1999; Ulloa et al., 2005). The allotetraploid species are believed to have evolved about 1.1–1.9 million years ago by polyploidization of diploid genomes closely related to the A1 genome of *Gossypium herbaceum* L. or A2 genome of *G. arboreum* L. (2*n* = 2*x* = 26); and the D5 genome of *G. raimondii* L. (2*n* = 2*x* = 26), and their domestication was result of extensive human selection (Wendel and Cronn, 2003). *G. hirsutum* L. (upland cotton, AD1 genome, 2*n* = 4*x* = 52) contributes around 95% of commercial cotton production due to its higher yield and wider environmental adaptation; whereas *G. barbadense* L. (Pima, Sea Island, and Egyptian cotton; AD2 genome, 2*n* = 4*x* = 52) contributes about 5% of total production and has superior fiber quality (Ulloa et al., 2005). *G. hirsutum* (GH after here) is high yielding with better quality fiber than diploid cottons, while *G. barbadense* (GB after here) is recognized for premium quality cotton fiber but low yield. Although reciprocal structure of these populations provides us a platform to investigate stable and a background specific QTL, only a few reciprocal mapping populations have been derived to study, segregation distortion regions and natural early leaf defoliation and introgression and mapping of fiber quality traits in cotton (Abdurakhmonov et al., 2005; Chandnani et al., 2017, 2018; Dai et al., 2017; Ulloa et al., 2017).

Cotton plant architectural traits are genetically complex, controlled by multiple genes (Guo et al., 2008; Luan et al., 2009; Song and Zhang, 2009; Lacape et al., 2013; Shang et al., 2015; Jia et al., 2016; Li et al., 2018, 2021; Ma et al., 2019; Wen et al., 2019). Plant height is the major determinant of plant architecture (Shang et al., 2015), with short height conferring higher lodging resistance and harvest index (Shang et al., 2015). The number of fruiting branches with one or two nodes is another important architectural trait and cotton fiber yield has been reported to be highly correlated with the number of fruiting sites (Grimes et al., 1969).

Like plant architectural traits, early maturity is of high importance for cotton cultivation. The longer maturity period of cotton than cereal crops makes a decision to dedicate farmland to a specific crop difficult (Li et al., 2013), often conferring a preference for short season or early maturing cotton cultivars. Similar to plant architectural traits, early maturity is complex and controlled by many QTLs (White, 1966; Yu et al., 2005; Dong et al., 2010; Li et al., 2013, 2021; Jia et al., 2016). Previously, most QTLs reported for plant architectural and maturity traits have been of ‘small’ effect (<10% PV) and henceforth warrant validation before being used in marker assisted breeding and improvement (Jia et al., 2016; Ma et al., 2019).

Here, at an advanced stage in the development of reciprocal advanced backcross populations from crosses between elite *Gossypium hirsutum* cultivar ‘*Acala Maxxa*’ (GH) and *G. barbadense* ‘*Pima S6*’ (GB), we undertook mapping of plant architectural traits, flowering time and maturity. Advanced backcrosses facilitate determination of more precise QTL effects and improve the detection of small effect QTLs by reducing background segregation.

## Materials and methods

### Plant material and phenotypic evaluation

To begin the development of advanced backcross population, F_1_ hybrids were generated by crossing elite cultivars where *Acala Maxxa* and *Pima S6* were each used as maternal parents to create initial F1 hybrids in GH and GB background, respectively. Subsequent backcrosses of F1 individuals to recurrent parents *Acala Maxxa* and *Pima S6*, respectively, produced reciprocal BC_1_ populations, with further backcrossing leading to BC_4_F_1_ populations. For recurrent backcrossing, backcross plants were used as maternal parent in both backgrounds. A total of 164 and 120 BC4F1 individuals in *Pima S6* and *Acala Maxxa* backgrounds, respectively, were evaluated for plant height (PH) and days to flowering (DOF) traits at the Univ. GA Plant Science Farm in Watkinsville, GA in 2012. In 2013, one replication of BC_4_F_2_ progeny row plots with 8–10 plants were again planted in Watkinsville and at the Coastal Plain Experiment Station, Tifton, GA in a completely randomized design, and later thinned to 5 plants. For the BC4F2 plants phenotyping, we used row plot average phenotype as that would most closely relate to individual BC4F1 plants that are segregating for very few and smaller genomic regions. A total of 8 traits were phenotyped including plant height (H), number of total branches (B), nodes with one branch (FBON), nodes with two or more branches (FBTN), days from planting to flowering (DOF), maturity (M), and residual flowering at the time of harvest (F). Maturity phenotype was visually scored in a range of 1–5, on the basis of percentage of cotton bolls that were open before harvest. Less than 25% opened bolls were given the score of 1, 2 for 25%, 3 for 50%, 4 for 75% and 5 when more than 75% of total bolls were open. H and B were recorded for three environments: Watkinsville 2012, Watkinsville 2013, Tifton 2013. DOF was recorded in two environments: Watkinsville 2012 and Watkinsville 2013. FBON, FBTN, M and F were recorded in two environments: Watkinsville 2013 and Tifton 2013. F was given phenotypic scales from 1 to 5 based on visual screening of residual flowering at the time of harvest. The F score of 1, 2, 3, 4 and 5 represented the genotype with ≤2, 2–4, 4–6, 7–9 and 10–12 residual flowers at the time of harvesting.

### Illumina library preparation, sequencing and mapping

DNA was extracted from the parents (*Acala maxxa* and *Pima s6*) and BC_4_F_1_ plants by rescaling the protocol described by Paterson et al. (1993) to a small scale version. GBS library preparation followed Andolfatto et al. (2011) with slight changes to optimize for cotton genome described in Chandnani et al. (2018). GBS library was sequenced for 150 cycles by single-end sequencing in an Illumina Miseq (Illumina) in by following the manufacturer’s recommendations. A total of 2.32 (*Pima s6*), 1.97 (*Acala maxxa*) and 0.41 (per BC_4_F_1_ individuals) million reads were input in TASSEL GBS pipeline. The TASSEL 4.0 GBS reference-based pipeline (Glaubitz et al., 2014) was run by using tetraploid GH (TM-1) as the reference genome (Zhang et al., 2015). Burrow Wheeler Alignment tool (BWA) and DiscoverySNPCallerPlugin was used to map and extract SNPs from GBS reads. Minimum read coverage was set to 5 to call SNP, and the minimum minor allele frequency (mnMAF) and site coverage (mnScov) of 0.01 and 0.2, respectively, were defined as SNP filtering parameters. To impute missing data, Fast Inbred Line Library ImputatioN (FILLIN) was used (Swarts et al., 2014). A total of 9,571 polymorphic SNPs were generated, from which 3,186 with less than 25% missing data were selected for genotyping.

### Marker-trait associations

A total of 3,186 and 3,026 polymorphic SNPs were used to genotype BC_4_F_1_ populations in GH and GB background, respectively. The genetic architecture (advanced backcross) and extreme segregation ratios of 15:1 in our population posed a limitation in the construction of a *de novo* genetic map. Therefore, for marker-trait associations, we used a physical reference map of GH (Zhang et al., 2015). Analysis of variance followed by stepwise regression was performed with significantly associated markers in ANOVA to calculate marker-trait associations. To control False Discovery Rate, *p* values were corrected by the Benjamini–Hochberg approach (Benjamini and Hochberg, 1995). Marker-trait associations with P_FDR_ < 0.01 were considered significant. ANOVA and Stepwise regression were performed in JMP SAS software. Minimum AICc criteria was used to select the stepwise regression model with highest phenotypic variation explained (SAS Institute Inc., North Carolina).

### Comparison of QTLs with previous reports and candidate gene identification

Physical co-ordinates of genetic markers flanking previously mapped QTLs, were obtained by blast with a TM-1 genome (Zhang et al., 2015) and/or the physical positions were obtained by published data from previous reports such as GWAS studies. If the QTLs identified in our study were within the QTL map interval or within 1.5 Mb (~2 cM) region of QTLs identified in previous reports, we declared it a case of co-localization. To identify gene IDs, only genes located within 50 Kb on each side of significantly associated SNP were obtained from the TM-1 genome (Zhang et al., 2015) in JBrowse (COTTONGEN).^1^ Putative functions of those genes were obtained from functional annotation data of GH (TM-1) from COTTNGEN.

## Results

### Phenotypic variation in reciprocal populations

GB parent Pima S6 had significantly higher (*p* < 0.05) values than GH parent *Acala Maxxa* for B, H, F, FBON and FBTN but not for M and DOF (Supplementary Table S1). In the BC_4_F_1_ population, for GB chromosomal segments introgressed into GH background, B, H, F, M, FBON and DOF showed normal distribution (skewness <2, kurtosis <2; Supplementary Table S1). In GB background with GH chromosomal segments introgressed, all traits showed normal distribution (skewness <2, kurtosis <2; Supplementary Table S1). Analysis of Variance revealed significance differences among genotypes for H, F, M, DOF in GH background, and for H, F and DOF in GB background, making these traits suitable for QTL mapping (Supplementary Table S2).

### Heritability and correlation among traits

Narrow sense heritability was calculated for PH and DOF traits (Supplementary Table S4). Heritability for architectural traits was low and in agreement with previous reports for cotton. Trait H had a significant positive correlation with B, FBON and FBTN but was negatively correlated with DOF in both backgrounds (Table 1). Trait M was negatively correlated with DOF in both backgrounds and positively correlated with F in GH background but not significantly in GB background. FBON and FBTN were significantly positively correlated with B in both backgrounds as expected.

**Table 1:** Correlation among plant architectural, flowering and maturity traits in reciprocal backgrounds.

		**GB background**						
		DOF	B	H	F	M	FBON	FBTN
**GH background**	DOF	1	−0.24^**^	−0.21^**^	0.04	−0.23^**^	−0.25^**^	−0.16
	B	−0.17	1	0.61^***^	0.03	0.05	0.79^***^	0.55^***^
	H	−0.18	0.67^***^	1	0.05	−0.07	0.47^***^	0.33^***^
	F	0.14	−0.1	0.05	1	0.1	0.01	0.12
	M	−0.29^**^	0.05	0.09	0.26^**^	1	0.1	0.17^*^
	FBON	−0.09	0.66^***^	0.53^**^	−0.09	0.04	1	0.44^***^
	FBTN	−0.1	0.28^**^	0.17	0.03	−0.05	0.25^**^	1

*, ** and *** represent significance level of *p* < 0.05, *p* < 0.01, and *p* < 0.001 respectively.

### Genomic distribution of SNPs and QTLs identified in reciprocal populations

**Figure 1:**
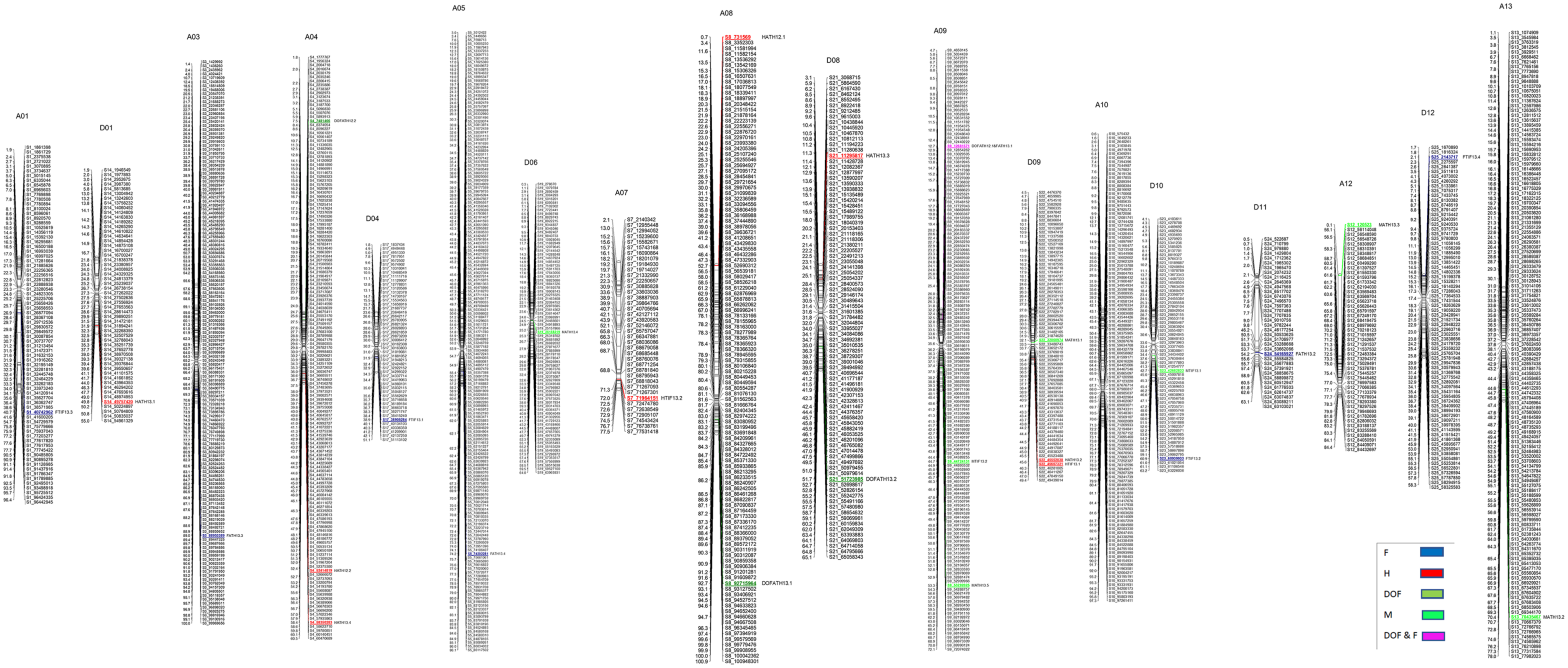
Chromosomal positions of SNP markers identified as QTLs for plant architectural, flowering and early maturity traits in an interspecific population in *Gossypium hirsutum* background introgressed with *Gossypium barbadense* chromosomal segments. QTLs for plant height (H) with red, days to flowering (DOF) with green, maturity (M) with light green, residual flowering (F) with blue, and residual flowering and days to flowering (F&DOF) were highlighted with magenta colors.

A total of 3,126 SNPs were used for QTL mapping in GH background where SNP number per chromosome varied from 47 for D01 to 218 for A05 chromosomes (Supplementary Table S3) and on an average 120 SNPs per chromosome were employed. In GB background, a total number of SNPs per chromosome varied from 42 to 216 on chromosome D11 and A05, respectively (Supplementary Table S3). In GH background, a total of 27 QTLs were identified based on segregation of introgressed GB chromosome segments (Figure 1). Most QTLs were of small effect (PV < 10%), explaining 4.8 to 12% of phenotypic variation (Table 2). The highest number of QTLs (8) were identified for residual flowering and height, whereas the date of flowering (DOF) trait was associated with the lowest number of QTL (4). The number of QTLs with positive effect allele introgressed from GB into GH background (21) was higher than the number of QTLs with negative effects (6).

**Table 2:** QTLs identified for DOF, F, H and M traits in *G. hirsutum* background.

**Trait**	**Environment**	**QTL name**	**Marker**	**Effect**	**PV%**	**PFDR**	**Direction**
DOF	2012 Athens	DOFATH12.1	S9_12681571	4.70	10.7	0.0004	Acala Maxxa
		DOFATH12.2	S4_7481466	4.77	12.0	0.0010	Acala Maxxa
	2013 Athens	DOFATH13.1	S8_92715964	8.18	5.7	0.0003	Acala Maxxa
		DOFATH13.2	S21_51723985	7.98	5.9	0.0003	Acala Maxxa
F	2013 Athens	FATH13.1	S9_12681571	0.54	7.8	0.0005	Acala Maxxa
		FATH13.2	S24_54165927	0.41	5.0	0.0007	Acala Maxxa
		FATH13.3	S3_88953389	0.58	5.1	0.0007	Acala Maxxa
		FATH13.4	S5_74202281	−0.39	5.8	0.0018	Pima S6
	2013 Tifton	FTIF13.1	S17_38905284	−0.34	6.1	0.0005	Pima S6
		FTIF13.2	S23_60939933	0.38	6.6	0.0019	Acala Maxxa
		FTIF13.3	S1_40742962	0.66	6.4	0.0019	Acala Maxxa
		FTIF13.4	S25_2143717	0.45	5.9	0.0057	Acala Maxxa
H	2012 Athens	HATH12.1	S8_731569	10.98	6.0	0.0050	Acala Maxxa
		HATH12.2	S4_52414819	6.28	8.9	0.0098	Acala Maxxa
	2013 Athens	HATH13.1	S14_49757420	−8.41	4.8	0.0023	Pima S6
		HATH13.2	S22_45552038	13.95	6.0	0.0023	Acala Maxxa
		HATH13.3	S21_11295817	−18.49	6.2	0.0026	Pima S6
		HATH13.4	S4_58350283	10.15	6.3	0.0036	Acala Maxxa
	2013 Tifton	HTIF13.1	S22_45607321	12.16	7.3	0.0006	Acala Maxxa
		HTIF13.2	S7_71964151	18.41	9.1	0.0011	Acala Maxxa
M	2013 Athens	MATH13.1	S22_32060974	−0.74	5.2	0.0004	Pima S6
		MATH13.2	S13_70435467	0.93	7.5	0.0029	Acala Maxxa
		MATH13.3	S12_120523	1.37	7.2	0.0029	Acala Maxxa
		MATH13.4	S19_25158319	1.48	9.0	0.0029	Acala Maxxa
		MATH13.5	S9_53292025	−1.15	7.8	0.0090	Pima S6
	2013 Tifton	MTIF13.1	S23_42682833	0.40	7.1	0.0010	Acala Maxxa
		MTIF13.2	S9_44724131	0.53	5.7	0.0010	Acala Maxxa

**Figure 2:**
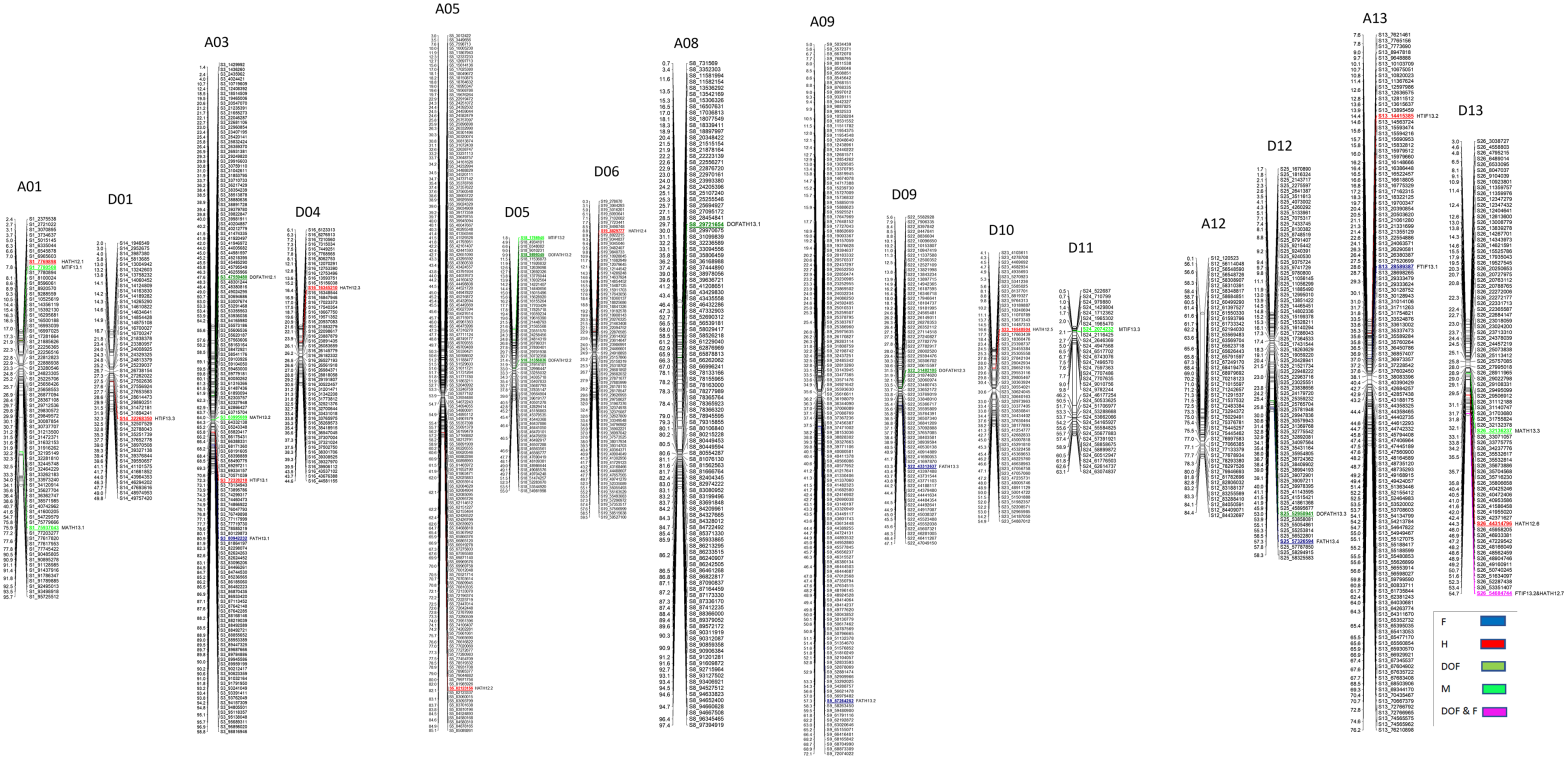
Chromosomal positions of SNP markers identified as QTLs for plant architectural, flowering and early maturity traits in an interspecific population in *Gossypium barbadense background* introgressed with *Gossypium hirsutum* chromosomal segments. QTLs for plant height (H) with red, days to flowering (DOF) with green, maturity (M) with light green, residual flowering (F) with blue, and residual flowering and days to flowering (F&DOF) were highlighted with magenta colors.

A total of 22 QTLs were identified in GB background, based on segregation of introgressed GH chromosome segments (Figure 2). Similar to GH background, all QTLs identified in this background were of small effect explaining from 1.8 to 9.3% of phenotypic variation (Table 3). Height was associated with the highest number of QTLs (10), whereas other traits had the same number (6) of QTLs. In contrast to GH background but consistent with the reciprocal nature of the study populations, >50% of QTLs (12) had negative effects alleles introgressed from GH into GB background across all traits (*p* < 0.05).

**Table 3:** QTLs identified for DOF, F, and H traits in *G. barbadense* background.

**Trait**	**Environment**	**QTL name**	**Marker**	**Effect**	**PV%**	**PFDR**	**Direction**
DOF	2012 Athens	DOFATH12.1	S3_47559480	5.97	7.3	0.0009	Pima S6
		DOFATH12.2	S18_9899345	4.35	5.9	0.0009	Pima S6
		DOFATH12.3	S22_31482105	4.32	2.6	0.0009	Pima S6
	2013 Athens	DOFATH13.1	S8_29721654	−5.97	6.6	0.0052	Acala maxxa
		DOFATH13.2	S18_31356836	6.49	4.1	0.0048	Pima S6
		DOFATH13.3	S25_52950941	6.17	2.6	0.0052	Pima S6
F	2013 Athens	FATH13.1	S3_80942232	−0.93	3.9	0.0001	Acala maxxa
		FATH13.2	S9_57264252	−0.37	4.2	0.0001	Acala maxxa
		FATH13.3	S22_43313637	−0.80	2.6	0.0001	Acala maxxa
		FATH13.4	S25_57326594	−0.52	9.3	0.0000	Acala maxxa
	2013 Tifton	FTIF13.1	S13_28589387	−0.48	3.9	0.0051	Acala maxxa
		FTIF13.2	S26_54684744	−0.68	2.4	0.0051	Acala maxxa
H	2013 Tifton	HTIF13.1	S3_72328218	14.85	2.8	0.0041	Pima S6
		HTIF13.2	S13_14415385	−12.09	5.8	0.0060	Acala maxxa
		HTIF13.3	S14_32268350	−17.34	3.8	0.0041	Acala maxxa
	2012 Athens	HATH12.1	S1_7769898	−4.25	5.1	0.0044	Acala maxxa
		HATH12.2	S5_82123156	4.53	2.3	0.0044	Pima S6
		HATH12.3	S16_16345210	4.25	1.8	0.0044	Pima S6
		HATH12.4	S19_8829777	−2.44	4.1	0.0044	Acala maxxa
		HATH12.5	S23_16548504	−6.06	3.7	0.0044	Acala maxxa
		HATH12.6	S26_44314796	6.38	2.7	0.0044	Pima S6
		HATH12.7	S26_54684744	7.81	5.2	0.0015	Pima S6

### QTLs identified for individual traits

#### Plant height

A total of 8 QTLs on 6 chromosomes were identified for plant height based on segregation of introgressed GB chromosome segments in GH background (Table 2). At two of 8 QTLs, alleles introgressed from GB into GH background reduced H whereas six of them increased H. Chromosomes from the A_t_ and D_t_ subgenomes each held 4 H QTLs. In GB background, a total of 10 QTLs were identified on 9 chromosomes (Table 3) based on segregation of introgressed GH chromosome segments. At 5 (50% of) QTLs, GH alleles introgressed into GB background reduced H and explained 3.7 to 5.8% of phenotypic variation. Six QTLs were on A_t_ subgenome chromosomes, and 4 on D_t_ chromosomes.

#### Residual flowering

In GH background, a total of 8 QTLs were identified based on segregation of introgressed GB chromosome segments, on 8 chromosomes (Table 2). Only 2 of 8 QTLs showed that residual flowering was decreased by GB allele introgressed into GH background and explained 5 to 7.8% of phenotypic variation. Equal numbers of QTLs were on A_t_ and D_t_ subgenome chromosomes. A total of 6 QTLs were identified in GB background based on segregation of introgressed *GH* chromosome segments, on 6 chromosomes (Table 3). At all of the QTLs, introgressed GH alleles had favorable effect (decreased residual flowering) on trait. These QTLs explained phenotypic variation ranging from 2.4 to 9.3%. Equal numbers of QTLs were identified on both subgenomes.

#### Days to flowering

A total of four DOF QTLs were identified based on segregation of introgressed GB chromosome segments in GH background, on chromosome A04, A08, A09 and D08. For all QTLs, the introgressed GB allele delayed flowering (Table 2). Phenotypic variation explained by these QTLs varied from 5.7–12% and 2 of 4 QTLs explained more than 10%. Only one out of 4 QTLs was on a D_t_ subgenome chromosome. A total of 6 QTLs were identified in GB background based on segregation of introgressed GH chromosome segments, on chromosomes A03, A08, D05 (2), D09 and D12. Phenotypic variation explained ranged from 2.6–7.3% (Table 3). At 5 of 6 QTLs, the introgressed GH allele delayed flowering - only on chromosome A08 did the GH allele accelerate flowering. Four of the 6 QTLs were on D_t_ subgenome chromosomes and 2 on A_t_ chromosomes.

#### Maturity

A total of 7 QTLs identified based on segregation of introgressed GB chromosome segments in GH background, were on 6 chromosomes (A09, A12, A13, D06, D09, D10; Table 2). Phenotypic variation explained ranged from 5.2–9.0% and at 5 of 7 QTLs, the GB allele increased maturity. Four of 7 QTLs were on A_t_ subgenome chromosomes and 3 on D_t_ chromosomes.

### Subgenomic origins of QTLs

There was no significant difference in the number of QTLs originating from A_t_ and D_t_ subgenomes, but GH and GB backgrounds favored different subgenomes. In GH background, a higher number (15) of QTLs were identified on A_t_ than D_t_ chromosomes (12); whereas in GB background the D_t_ subgenome held more QTLs (Table 4). The lack of significance of these differences and small number of architectural traits that segregated for QTLs limits one’s ability to draw conclusions about these findings.

**Table 4:** Subgenomic distribution of QTLs in *G. hirsutum* and *G. barbadense* background.

**Trait**	**GH background**		**GB Background**	
	**A**_**t**_	**D**_**t**_	**A**_**t**_	**D**_**t**_
Date of flowering (DOF)	3	1	2	4
Flowering (F)	4	4	3	3
Height (H)	4	4	4	6
Maturity (M)	4	3		
Total	15	12	9	13

### QTL clusters and QTLs with pleiotropic effects

Previous meta-analysis studies of QTLs in cotton have observed and defined QTL clusters and hotspots located in an average interval of 20 cM (Said et al., 2015). In our case, to define physical regions covering approximately 20 cM, we compared the genetic length to physical length of individual chromosomes [from a consensus genetic map by Blenda et al. (2012)] to calculate physical distance corresponding to 20 cM. Quantitative Trait Loci are known to be located nonrandomly or in clusters in genomes and we did observe QTLs for the same or different traits clustered within 20 cM windows. For GB chromosomal segments introgressed into GH background, a total of two clusters were observed on chromosomes A04 and A09 (Table 5). Cluster CHQTLA04.1 contained two QTLs for H and at both QTLs, the introgressed GB allele increased H. Cluster CHQTLA09.1 contained two QTLs for maturity—GB alleles introgressed into GH background showed positive effects (increased M) at one and negative effects at the other. For GH chromosomal segments introgressed into GB background, a total of 2 clusters were observed on chromosomes A03 and D12 with 2 QTLs in each. Cluster CBQTLA03.1 contained one QTL each for H and F, and the introgressed GH allele increased height and reduced residual flowering. Cluster CBQTLD12.1 harbored one QTL each for DOF and F, and the introgressed GH allele delayed DOF and reduced residual flowering at maturity.

**Table 5:** QTL clusters in *G. hirsutum* and *G. barbadense* background.

Background	Cluster	Chromosome	QTLs	Physical interval (Mb)
GH	CBQTLD012.1	A04	HATH12.2, HATH13.4	52.4–58.3
	CHQTLA09.1	A09	MTIF13.2, MATH13.5	44.7–53.3
GB				
	CBQTLA03.1	A03	HTIF13.1, FATH13.1	72.3–80.9
	CBQTLD012.1	D12	DOFATH13.3, FATH13.4	52.9–57.3

Putative pleiotropic effects of QTLs were only observed for one case in each background. For GB chromosomal segments introgressed into GH background, on chromosome A09 the same SNP marker was associated with DOF and F, and the GB allele increased the value of both traits. For GH chromosomal segments introgressed into GB background, F and H were associated with a single SNP on chromosome D13, and the introgressed GH allele increased H but decreased F.

### Common QTLs in both the backgrounds with opposite effect

Only one common QTL was identified in both backgrounds, on chromosome A03 for trait F, and the GB allele increased, and GH allele decreased F.

### Plant height, flowering time and early maturity related genes near QTLs validated in previous reports

A total of 7 QTLs, (4 in GH background and 3 in GB) co-localized (Table 6) with plant architectural and early maturity trait QTLs reported previously (Jia et al., 2016; Li et al., 2018, 2021; Ma et al., 2019; Wen et al., 2019). A total of 26 genes (12 in GH and 14 in GB background) were identified (Table 7) within 100 Kb of QTL SNP (50 Kb on each side of the SNP). Due to unavailability of an *Acala Maxxa* reference genome sequence and annotation, we used well annotated TM-1 as reference genome. For plant height, in GH background we identified genes related to 50s ribosomal protein, AMP-binding domain protein, microsomal signal peptide and glucose 1 phosphate within 100 Kb of HATH13.1 QTL on chromosome D01. On chromosome A07, we identified adenylate kinase, 1-phosphate adenylyltransferase, small GTPase Rab1 and related to peptidase inhibitor I9 genes. In GB background, for plant height only one gene related to AT hook DNA binding motif was identified on chromosome D01. For early maturity, we identified, genes related to 50S ribosomal protein and pentatricopeptide (PPR) repeat families in GH background on chromosome A13. In GB background, we identified Serine–threonine/tyrosine-protein kinase, ankyrin repeats, small GTPase Rab1, Mitochondrial termination factor repeats, Cyclin and N-terminal domain like genes near flowering time QTL on chromosome D09. For residual flowering QTLs in GH background, protein tyrosine kinase, Armadillo-type fold and phosphoribosyl pyrophosphate synthase like genes were identified on chromosome A03 in GH background; and in GB background, DnaJ domain signature, Leucine-rich repeat, SDS22-like, Methionine aminopeptidase-1, Adenosine and AMP deaminase and Acyl-CoA dehydrogenase like genes were identified on chromosome D09.

**Table 6:** QTLs co-localizated with physical regions of plant architectural and early maturity trait QTLs identified in previous reports.

	**Trait**	**QTL name**	**Marker**	**Effect**	**PV%**	**PFDR**	**Direction**	**Validation with previous studies**
**GB Background**	F	FATH13.3	S3_88953389	0.58	5.1	0.0007	Acala Maxxa	First boll opening, GWAS (Li et al., 2021)
	H	HATH13.1	S14_49757420	−8.41	4.8	0.0023	Pima S6	PH, GH X GB (Jia et al., 2016; Ma et al., 2019)
	H	HTIF13.2	S7_71964151	18.41	9.1	0.0011	Acala Maxxa	First boll opening, GWAS (Li et al., 2021)
	M	MATH13.2	S13_70435467	0.93	7.5	0.0029	Acala Maxxa	fruit spur branch number, GWAS (Wen et al., 2019)
**GB Background**	DOF	DOFATH12.3	S22_31482105	4.32	2.6	0.0009	Pima S6	PH, GWAS (Wen et al., 2019)
	F	FATH13.3	S22_43313637	−0.8	2.6	0.0001	Acala maxxa	Flower and boll period, GWAS (Li et al., 2018)
	H	HTIF13.3	S14_32268350	−17.3	3.8	0.0041	Acala maxxa	PH, GH X GB (Jia et al., 2016; Ma et al., 2019)

**Table 7:** Genes/families identified in within 100 Kb of significantly associated SNPs that co-localized with previously identified QTLs.

	**Trait**	**QTL name**	**Gene_ID**	**Gene/families**
**GB Background**	F	FATH13.3	Gh_A03G1269	Protein tyrosine kinase
			Gh_A03G1268	HEAT repeat associated with sister chromatid cohesion
			Gh_A03G1270	Phosphoribosyl pyrophosphate synthase
	H	HATH13.1	Gh_D01G1585	Adenylate kinase
			Gh_D01G1584	AMP-binding enzyme
			Gh_D01G1587	Microsomal signal peptidase
			Gh_D01G1586	50S ribosomal protein
			Gh_A07G1759	Glucose-1-phosphate adenylyltransferase
	H	HTIF13.2	Gh_A07G1757	Peptidase inhibitor I9
			Gh_A07G1758	Small GTPase Rab1 family
	M	MATH13.2	Gh_A13G1387	50S ribosomal protein
			Gh_A13G1386	Pentatricopeptide (PPR) repeat
**GB Background**	DOF	DOFATH12.3	Gh_D09G0732	Serine/Threonine protein kinases active-site signature.
			Gh_D09G0737	ankyrin repeats
			Gh_D09G0735	small GTPase Rab1 family profile.
			Gh_D09G0736	Mitochondrial termination factor repeats
			Gh_D09G0734	domain present in cyclins, TFIIB and Retinoblastoma
	F	FATH13.3	Gh_D09G1569	Acyl-CoA dehydrogenase, N-terminal domain
			Gh_D09G1570	Glutaredoxin domain profile.
			Gh_D09G1571	Adenosine and AMP deaminase signature.
			Gh_D09G1572	Small hydrophilic plant seed protein
			Gh_D09G1573	Methionine aminopeptidase-1 signature
			Gh_D09G1574	DnaJ domain signature
			Gh_D09G1575	Thiamine pyrophosphate enzyme, N-terminal TPP binding domain
			Gh_D09G1576	Leucine-rich repeat, SDS22-like subfamily
	H	HTIF13.3	Gh_D01G1264	AT hook DNA binding motif

## Discussion

Plant architectural and maturity traits are key factors that have significant impact on yield and economic value of cotton. Early maturity traits have been reported to be negatively correlated with lint yield, but are sometimes required for efficient farmland use in crop rotations (Song et al., 2005). Complexities of plant architectural traits make improvement in these traits by conventional breeding difficult—trait dissection and DNA marker identification can improve understanding of these traits. This study shows that both parents have contributed positive and negative effect alleles for H, F, M, and DOF. These QTLs provide opportunities to manipulate architectural traits and develop cultivars with improved fiber quality and increased yield in both GH and GB backgrounds.

### Background specific new allelic combinations

Despite no significant difference between GH and GB parents for DOF, we could identify loci in both backgrounds that increased and decreased maturity, which indicate new genetic combinations created by introgression in both backgrounds. Despite the GB parent being inferior for traits H and F, GB chromosomal segments introgressed into GH background conferred beneficial effects. Contributions of such ‘cryptic’ favorable alleles from inferior parents have been reported previously for many fiber property traits and architectural traits in cotton (Zhang et al., 2011).

### Correlation among traits and QTL clustering

A significant positive correlation between plant height and number of fruiting branches was consistent with previous reports in cotton (Song and Zhang, 2009). Similarly, days to flowering and maturity were negatively correlated in both backgrounds. Although not statistically significant, curiously, plant height and maturity were nominally positively correlated in GH background and negatively correlated in GB background. Positive correlation between DOF and F in GH background agreed with clustering of positive effect QTLs for these traits on chromosome A09. In GB background introgressed with GH chromosomal segments, despite clustering of positive effect H and negative effect F QTLs, non-significant but nominally positive correlation existed between these traits. Conflicts between trait correlation and QTL clustering suggests the presence of unidentified QTLs in the background, or that these traits are more affected by environment than by genetics. A portion of flowering related early maturity QTLs in our study co-localized with plant height QTLs from previous studies and vice versa. These results agreed with previous findings of strong correlation in plant height and early maturity traits in cotton populations (Jia et al., 2016).

### Lack of reciprocal QTLs

An unexpected result was that in very few cases, indeed only one, QTLs at corresponding locations in the reciprocal backgrounds showed the expected reciprocal (opposite) phenotypic effects. One major reason for this unexpected finding could be that a significant proportion (30%) of QTLs for plant architectural traits have been reported to be epistatic. Low heritability of these traits support this argument (Song and Zhang, 2009), as does our observation of many cryptic QTLs that are inconsistent with parental phenotypes. Other possible reasons that might explain limited correspondence in results from reciprocal backgrounds are comparatively lower coverage of donor alleles at these loci in one of the backgrounds. To test this hypothesis, we performed chi squared tests to assess donor allele coverage at QTL loci. Donor allele coverage was significantly low for 9 QTL loci in GH background and for 4 QTL loci in GB background and this might be a reason that reciprocal QTLs were not detected at some expected loci. Another factor could be the small effect of most of the identified QTLs, increasing the likelihood that one or both members of a reciprocal pair elude detection (Broman et al., 2003).

### Smaller introgression regions provide higher resolution for QTL mapping

Similarity of QTL locations with previous reports can strengthen the evidence supporting validity of QTLs. Interestingly, two plant height QTLs (HATH13.1 in GH background and HTIF13.3 in GB background) on chromosome D01 with contrasting effects in reciprocal backgrounds co-localized with single plant height QTL mapped in a (GH X GB) BC1F8 population (Jia et al., 2016; Ma et al., 2019). The much larger QTL interval in the BC1F8 population (30.7–53 Mb), could contain two opposite-effect QTLs detected in our advanced backcross populations at 32.2 and 49.7 Mb positions. These results corroborate how a smaller number and size of introgressions improves the resolution and power to detect small effect QTLs. Ma et al. (2019) identified a putative negative regulator of cotton plant height near one of the two QTL (HATH13.1) that co-localized with the plant height QTL from Ma et al. (2019). Near the second QTL detected in our study in the co-localized region, we identified an AT hook DNA binding motif like gene, reported to enhance plant height upon overexpression in *Zoysia japonica* (Jeong et al., 2020) and reduced plant height in a CRISPR/Cas9 mutant of *sihmg3* (protein that contains 4 AT hook repeats) in tomato (Li et al., 2022).

### Putative candidate genes for plant height, flowering time and early maturity in cotton

Putative genes families near early maturity, flowering time and plant height QTLs in our study have been reported to have similar functions in Arabidopsis and other crops. Putative candidate genes identified near plant height QTLs were related to adenylate kinase and ribosomal proteins. Adenylate kinases catalyze energy conversion reactions related to photosynthesis (Igamberdiev and Kleczkowski, 2001) and respiratory metabolism (Igamberdiev and Kleczkowski, 2003) and therefore, are critical for plant growth. Feng et al. (2012) discovered that *Arabidopsis* adenylate kinase 6 is essential for stem growth and a T-DNA mutant *akk6* showed shorter stem than wild type Arabidopsis. Further, recently, Zhang et al. (2021), identified that an adenylate kinase *OsAK3* involved in brassinosteroid signaling, positively regulates rice plant height as an *osak3* mutant exhibited dwarf phenotype with shorter grain length. Ribosomal proteins such as large subunit protein 3B (RPL3B) encoded by the RML1 gene positively regulate plant height in rice (Zheng et al., 2016).

A gene related to Pentatricopeptide (PPR) repeat family was identified near early maturity QTL in GH background. PRECOCIOUS1 (POCO1), a mitochondrial pentatricopeptide repeat gene, has been reported to regulate flowering time in Arabidopsis, in which a T-DNA insertion mutation exhibited earlier flowering than wild type (Emami and Kempken, 2019). Additionally, expression of a MADS-box transcription factor, FLOWERING LOCUS C (FLC, a flowering repressor), was downregulated in the mutant Arabidopsis plants (Emami and Kempken, 2019). Genes related to Serine/Threonine protein kinase family, in the vicinity of flowering time QTLs in GB background, have been implicated in Brassinosteroid signaling and flowering time in plants (Oh et al., 2009). Quite a few studies have reported that mutation in brassinosteroid insensitive and biosynthesis gene resulted in late flowering (Chory et al., 1991; Azpiroz et al., 1998; Li et al., 2010). Identification of a DnaJ-domain protein near residual flowering QTL correlated with a study (Park et al., 2011) that suggested that *EMBRYONIC FLOWER* (*EMF*) *1* (considered a floral repressor) interaction protein EIP9 (a DnaJ type protein) regulates flowering time in Arabidopsis. Early flowering was observed in *eip9* mutants and late flowering upon overexpression (Park et al., 2011).

### Nature of QTLs identified in our population and their importance in cotton breeding

QTL mapping in advanced backcross generation detects both dominant and additive allele effects, however, does not permit us to distinguish them. Each of these are important to cotton improvement. Main effect QTLs with dominance have been reported to contribute to heterosis for plant architectural traits in cotton (Shang et al., 2016). Heterosis have been reported to be the major driving factor for development of hybrid cultivars (which are widespread in some countries) and complex trait improvement (Randhawa and Singh, 1994; Wu et al., 2004). The focus of this study was to identify quantitative loci associated with architectural traits, reducing background noise and increasing resolution of introgression regions. Complex traits are often governed by multiple small effect QTLs that are difficult to discern—our approach identified some such small effect QTLs that can be important in understanding genetic control of complex architectural traits. Moreover, intensive selection and breeding of elite cotton cultivars may have resulted in fixation of major effect loci, with further advances in breeding relying on small effect loci. Although small effect loci have minimal contributions individually, some can also influence the effect of other loci by interaction (Isobe et al., 2007; Jannink, 2007; Ravi et al., 2011). Thus, while major effect QTLs are a natural priority in marker assisted selection, pyramiding of small effect loci can further contribute to cotton breeding for architectural (or other) trait improvement.

## Conclusion

Molecular dissection of plant architectural and flowering traits related to early maturity in this study validate the complex nature of these traits and illustrate the merit of high-resolution mapping populations with smaller size and fewer introgressions. In some cases, our extensive backcrossing indicated multiple compensatory QTLs within genomic regions reported to have only single QTL for the trait. Validation of QTLs with previous biparental and GWAS populations support the candidacy of QTLs reported in this study. In addition to its merit for quantitative trait mapping and as a resource to study evolutionary biology, this population serves as potential germplasm for pre-breeding programs to develop cotton lines beneficial for plant architectural and fiber quality traits in each of the two most widely cultivated species of cotton.

## Data availability statement

The raw sequencing data presented in the study are deposited in the bioproject PRJNA861271 in NCBI repository, accession number SAMN29903056.

## Author contributions

RC conducted backcrossing, developed mapping population, phenotyping, genotyping, data analysis, and QTL mapping, and wrote the manuscript. CK and TS helped in illumine library preparation and sequencing. HG helped with sequence alignment and SNP HapMap generation. JW helped with missing data imputation. ZZ helped in development of reciprocal F1 population. JP, JA, PC, DH, and SK helped with planting and phenotyping. AP conceived and supervised the project, acquired funding, and contributed to manuscript writing and revision. All authors contributed to the article and approved the submitted version.

## Funding

Authors sincerely thank and appreciate the National Science Foundation (Grant number DBI 0817707) for funding of the project.

## Conflict of interest

The authors declare that the research was conducted in the absence of any commercial or financial relationships that could be construed as a potential conflict of interest.

## Publisher’s note

All claims expressed in this article are solely those of the authors and do not necessarily represent those of their affiliated organizations, or those of the publisher, the editors and the reviewers. Any product that may be evaluated in this article, or claim that may be made by its manufacturer, is not guaranteed or endorsed by the publisher.

## Footnotes

^1^ https://www.cottongen.org/data/download

## Abbreviations

qtl: Quantitative trait loci
GH: *Gossypium hirsutum*
GB: *Gossypium barbadense*
H: Plant height
B: Number of total branches
FBON: Nodes with one branch
FBTN: Nodes with two or more branches
DOF: Days from planting to flowering
M: Maturity
F: Residual flowering at the time of harvest
pv: Phenotypic variation
