# Supplementary material for: Identification of small effect quantitative trait loci of plant architectural, flowering, and early maturity traits in reciprocal interspecific introgression population in cotton
Source: Front Plant Sci. 2022 Aug 18;13:981682. doi: 10.3389/fpls.2022.981682 (PMC9433993; doi:10.3389/fpls.2022.981682)
Supplement: Supplementary file 1 [file Data_Sheet_1.docx]

Table S1. Descriptive statistics of plant architectural, flowering and maturity traits in *G. hirsutum* and *G. barbadense* backgrounds

|  |  | A  DOF | AVG  B | AVG  PH | AVG  F | AVG  M | AVG  FBON | AVG  FBTN |
| --- | --- | --- | --- | --- | --- | --- | --- | --- |
| Parental genotypes | GH (Acala Maxxa) | 57.58 | 12.5 | 77.61 | 3.83 | 3.42 | 10.67 | 0.33 |
|  | GB(Pima S6) | 56.41 | 16.22 | 99.94 | 4.67 | 3.75 | 12.33 | 1.58 |
|  | GB-GH | 0.300 | 0.010 | <0.0001 | 0.001 | 0.260 | 0.030 | 0.003 |
| GH background | Mean | 58.94 | 13.7 | 67.41 | 3.98 | 3.03 | 11.78 | 0.58 |
|  | *SD* | 4.08 | 1.55 | 9.05 | 0.39 | 0.62 | 1.58 | 0.64 |
|  | Skewness | 0.75 | 0.64 | 0.33 | 0.29 | 0.12 | -0.1 | 1.62 |
|  | Kurtosis | -0.19 | 0.82 | -0.15 | -0.17 | -0.38 | -0.12 | 3.62 |
|  | CV | 6.92 | 11.35 | 13.43 | 9.83 | 20.54 | 13.46 | 109.66 |
|  | Min | 53 | 10 | 48.7 | 3 | 1.5 | 7 | 0 |
|  | Max | 71.5 | 18.7 | 91 | 5 | 4.5 | 16 | 3 |
| GB background | Mean | 60.82 | 15.79 | 80.49 | 4.82 | 3.49 | 12.99 | 1.59 |
|  | *SD* | 4.22 | 1.7 | 8.22 | 0.4 | 0.6 | 1.65 | 0.87 |
|  | Skewness | 0.56 | 0.59 | 0.2 | -1.94 | -0.58 | 0.36 | 0.67 |
|  | Kurtosis | -0.22 | 0.75 | 0.4 | 2.61 | -0.4 | 0.15 | 0.35 |
|  | CV | 6.94 | 10.77 | 10.21 | 8.37 | 17.22 | 12.71 | 54.72 |
|  | Min | 53 | 12 | 57 | 3 | 2 | 9 | 0 |
|  | Max | 75 | 22 | 104 | 5 | 5 | 18 | 4 |

Table S2. Analysis of variance for plant architectural, flowering and maturity traits in reciprocal backgrounds

|  |  | GH background | | | | | GB background | | | | | |
| --- | --- | --- | --- | --- | --- | --- | --- | --- | --- | --- | --- | --- |
| Trait | S | DF | SS | MS | F Val | Pr > F | DF | SS | MS | F Val | Pr > F |  |
| B | G | 121 | 1002.6 | 8.29 | 1.38 | 0.16 | 165 | 1568.3 | 9.50 | 1.58 | 0.07 |  |
|  | E | 2 | 1638.2 | 819.10 | 136.14 | <.0001 | 2 | 3751.4 | 1875.68 | 311.75 | <.0001 |  |
|  | G*E | 242 | 2010.1 | 8.31 | 1.38 | 0.15 | 330 | 2834.5 | 8.59 | 1.43 | 0.12 |  |
| H | G | 121 | 44341.8 | 366.46 | 2.89 | 0.00 | 165 | 39728.7 | 240.78 | 1.90 | 0.02 |  |
|  | E | 2 | 22457.9 | 11228.93 | 88.52 | <.0001 | 2 | 385332.3 | 192666.15 | 1518.78 | <.0001 |  |
|  | G*E | 242 | 55567.1 | 229.62 | 1.81 | 0.03 | 330 | 90842.6 | 275.28 | 2.17 | 0.01 |  |
| DOF | G | 121 | 4046.6 | 33.44 | 3.10 | 0.00 | 165 | 6047.2 | 36.65 | 3.40 | 0.00 |  |
|  | E | 1 | 2659.3 | 2659.28 | 246.61 | <.0001 | 1 | 2344.1 | 2344.14 | 217.39 | <.0001 |  |
|  | G*E | 121 | 3316.2 | 27.41 | 2.54 | 0.01 | 161 | 6100.5 | 37.89 | 3.51 | 0.00 |  |
| F | G | 121 | 42.2 | 0.35 | 1.90 | <0.05 | 165 | 61.4 | 0.37 | 2.03 | 0.03 |  |
|  | E | 1 | 11.7 | 11.67 | 63.66 | <.0001 | 1 | 0.0 | 0.00 | 0.02 | 0.90 |  |
|  | G*E | 121 | 44.5 | 0.37 | 2.01 | 0.04 | 165 | 51.2 | 0.31 | 1.69 | 0.09 |  |
| M | G | 121 | 99.3 | 0.82 | 1.97 | 0.04 | 165 | 101.8 | 0.62 | 1.48 | 0.15 |  |
|  | E | 1 | 8.7 | 8.67 | 20.80 | 0.00 | 1 | 14.3 | 14.35 | 34.43 | <.0001 |  |
|  | G*E | 121 | 77.5 | 0.64 | 1.54 | 0.13 | 165 | 84.6 | 0.51 | 1.23 | 0.30 |  |
| FBON | G | 121 | 616.2 | 5.09 | 1.49 | 0.15 | 165 | 958.3 | 5.81 | 1.70 | 0.08 |  |
|  | E | 1 | 0.0 | 0.00 | 0.00 | 0.97 | 1 | 42.4 | 42.37 | 12.40 | 0.00 |  |
|  | G*E | 121 | 450.9 | 3.73 | 1.09 | 0.43 | 165 | 880.5 | 5.34 | 1.56 | 0.12 |  |
| FBTN | G | 121 | 108.5 | 0.90 | 0.94 | 0.61 | 165 | 237.4 | 1.44 | 1.50 | 0.14 |  |
|  | E | 1 | 0.7 | 0.68 | 0.71 | 0.41 | 1 | 6.5 | 6.48 | 6.77 | 0.02 |  |
|  | G*E | 121 | 72.4 | 0.60 | 0.62 | 0.94 | 165 | 183.1 | 1.11 | 1.16 | 0.37 |  |

Table S3. Genome wide distribution of SNPs in reciprocal backgrounds

| Chromosome | No of SNPs (GH background) | No of SNPs (GB background) |
| --- | --- | --- |
| A01 | 109 | 104 |
| A02 | 163 | 159 |
| A03 | 195 | 192 |
| A04 | 199 | 180 |
| A05 | 218 | 216 |
| A06 | 187 | 183 |
| A07 | 58 | 57 |
| A08 | 166 | 159 |
| A09 | 215 | 213 |
| A10 | 157 | 145 |
| A11 | 156 | 156 |
| A12 | 59 | 59 |
| A13 | 178 | 169 |
| D01 | 69 | 64 |
| D02 | 118 | 108 |
| D03 | 90 | 75 |
| D04 | 64 | 47 |
| D05 | 77 | 73 |
| D06 | 105 | 102 |
| D07 | 82 | 74 |
| D08 | 108 | 107 |
| D09 | 89 | 84 |
| D10 | 76 | 67 |
| D11 | 47 | 42 |
| D12 | 93 | 93 |
| D13 | 108 | 98 |
| Total | 3186 | 3026 |

Table S4. Narrow sense heritability of DOF and PH in both generations

|  | GH (background) | | GB (background) | |  |
| --- | --- | --- | --- | --- | --- |
| Trait | ATH | TIF | ATH | TIF |  |
| H | 0.1 | 0.14 | 0.16 | 0.13 |  |
| DOF | 0.22 | NA | NA | NA |  |
|  |  |  |  |  |  |
| NA represent either non availability of phenotyping data or no heritability between traits | | | | | |

| Table S5.1. Allele frequencies at QTL loci in GH background | | | | | |  |  |  |  |  |
| --- | --- | --- | --- | --- | --- | --- | --- | --- | --- | --- |
|  |  |  |  |  |  |  |  |  |  |  |
| Trait | Environment | QTL name | Marker | Effect | PV% | *A maxxa* | *Pima s6* | Major allele freq | Heterozygote freq |  |
| DOF | 2012 Athens | DOFATH12.1 | S9_12681571 | 4.7 | 10.7 | G | A | 90.8 | 8.3 |  |
|  |  | DOFATH12.2 | S4_7481466 | 4.77 | 12 | G | C | 90.8 | 4.2 |  |
|  | 2013 Athens | DOFATH13.1 | S8_92715964 | 8.18 | 5.7 | G | T | 92.5 | 4.2 |  |
|  |  | DOFATH13.2 | S21_51723985 | 7.98 | 5.9 | G | T | 90.8 | 6.7 |  |
| F | 2013 Athens | FATH13.1 | S9_12681571 | 0.54 | 7.8 | G | A | 90.8 | 8.3 |  |
|  |  | FATH13.2 | S24_54165927 | 0.41 | 5 | C | T | 92.5 | 5.8 |  |
|  |  | FATH13.3 | S3_88953389 | 0.58 | 5.1 | G | A | 95.0 | 4.2 |  |
|  |  | FATH13.4 | S5_74202281 | -0.39 | 5.8 | G | A | 87.5 | 11.7 |  |
|  | 2013 Tifton | FTIF13.1 | S17_38905284 | -0.34 | 6.1 | G | A | 67.5 | 26.7 |  |
|  |  | FTIF13.2 | S23_60939933 | 0.38 | 6.6 | A | C | 69.2 | 24.2 |  |
|  |  | FTIF13.3 | S1_40742962 | 0.66 | 6.4 | T | G | 91.7 | 6.7 |  |
|  |  | FTIF13.4 | S25_2143717 | 0.45 | 5.9 | A | C | 85.8 | 11.7 |  |
| H | 2012 Athens | HATH12.1 | S8_731569 | 10.98 | 6 | T | C | 93.3 | 3.3 |  |
|  |  | HATH12.2 | S4_52414819 | 6.28 | 8.9 | G | A | 90.0 | 8.3 |  |
|  | 2013 Athens | HATH13.1 | S14_49757420 | -8.41 | 4.8 | C | T | 79.2 | 14.2 |  |
|  |  | HATH13.2 | S22_45552038 | 13.95 | 6 | A | G | 83.3 | 12.5 |  |
|  |  | HATH13.3 | S21_11295817 | -18.49 | 6.2 | A | C | 92.5 | 5.8 |  |
|  |  | HATH13.4 | S4_58350283 | 10.15 | 6.3 | A | G | 92.5 | 5.0 |  |
|  | 2013 Tifton | HTIF13.1 | S22_45607321 | 12.16 | 7.3 | C | T | 76.7 | 15.0 |  |
|  |  | HTIF13.2 | S7_71964151 | 18.41 | 9.1 | A | G | 90.8 | 6.7 |  |
| M | 2013 Athens | MATH13.1 | S22_32060974 | -0.74 | 5.2 | T | G | 87.5 | 10.8 |  |
|  |  | MATH13.2 | S13_70435467 | 0.93 | 7.5 | T | C | 89.2 | 8.3 |  |
|  |  | MATH13.3 | S12_120523 | 1.37 | 7.2 | G | C | 86.7 | 4.2 |  |
|  |  | MATH13.4 | S19_25158319 | 1.48 | 9 | C | G | 95.0 | 4.2 |  |
|  |  | MATH13.5 | S9_53292025 | -1.15 | 7.8 | T | A | 92.5 | 5.8 |  |
|  | 2013 Tifton | MTIF13.1 | S23_42682833 | 0.4 | 7.1 | C | T | 79.2 | 18.3 |  |
|  |  | MTIF13.2 | S9_44724131 | 0.53 | 5.7 | G | A | 90.8 | 8.3 |  |
| Table S5.2. Allele frequencies at QTL loci in reciprocal backgrounds GB background | | | | | | | | | |  |
| Trait | Environment | QTL name | Marker | Effect | PV% | *Pima s6* | *A maxxa* | Major allele freq | Heterozygote freq | |
| DOF | 2012 Athens | DOFATH12.1 | S3_47559480 | 5.97 | 7.3 | T | C | 94.5 | 2.4 |  |
|  |  | DOFATH12.2 | S18_9899345 | 4.35 | 5.9 | G | A | 91.5 | 4.3 |  |
|  |  | DOFATH12.3 | S22_31482105 | 4.32 | 2.6 | C | T | 92.1 | 2.4 |  |
|  | 2013 Athens | DOFATH13.1 | S8_29721654 | -5.97 | 6.6 | T | A | 85.4 | 10.4 |  |
|  |  | DOFATH13.2 | S18_31356836 | 6.49 | 4.1 | G | A | 89.6 | 4.9 |  |
|  |  | DOFATH13.3 | S25_52950941 | 6.17 | 2.6 | G | A | 92.1 | 3.7 |  |
| F | 2013 Athens | FATH13.1 | S3_80942232 | -0.93 | 3.9 | C | T | 93.3 | 1.2 |  |
|  |  | FATH13.2 | S9_57264252 | -0.37 | 4.2 | C | A | 85.4 | 9.8 |  |
|  |  | FATH13.3 | S22_43313637 | -0.8 | 2.6 | T | C | 92.1 | 1.2 |  |
|  |  | FATH13.4 | S25_57326594 | -0.52 | 9.3 | G | A | 82.9 | 8.5 |  |
|  | 2013 Tifton | FTIF13.1 | S13_28589387 | -0.48 | 3.9 | T | A | 91.5 | 5.5 |  |
|  |  | FTIF13.2 | S26_54684744 | -0.68 | 2.4 | G | A | 86.0 | 2.4 |  |
| H | 2013 Tifton | HTIF13.1 | S3_72328218 | 14.85 | 2.8 | T | C | 94.5 | 3.0 |  |
|  |  | HTIF13.2 | S13_14415385 | -12.09 | 5.8 | G | A | 89.6 | 4.9 |  |
|  |  | HTIF13.3 | S14_32268350 | -17.34 | 3.8 | A | G | 87.2 | 3.7 |  |
|  | 2012 Athens | HATH12.1 | S1_7769898 | -4.25 | 5.1 | C | G | 87.2 | 8.5 |  |
|  |  | HATH12.2 | S5_82123156 | 4.53 | 2.3 | G | A | 85.4 | 6.1 |  |
|  |  | HATH12.3 | S16_16345210 | 4.25 | 1.8 | T | C | 81.7 | 6.1 |  |
|  |  | HATH12.4 | S19_8829777 | -2.44 | 4.1 | A | T | 54.9 | 35.4 |  |
|  |  | HATH12.5 | S23_16548504 | -6.06 | 3.7 | A | T | 94.5 | 2.4 |  |
|  |  | HATH12.6 | S26_44314796 | 6.38 | 2.7 | C | T | 92.1 | 1.2 |  |
|  |  | HATH12.7 | S26_54684744 | 7.81 | 5.2 | G | A | 86.0 | 2.4 |  |
